# A Case Study on the Impacts of Social Contexts on a Chinese English as a Foreign Language Learner’s L1 and L2 Identities Development

**DOI:** 10.3389/fpsyg.2021.772777

**Published:** 2022-01-06

**Authors:** Yuehai Xiao, Angel Zhao

**Affiliations:** ^1^Department of English, Hunan Normal University, Changsha, China; ^2^Beijing Normal University-Hong Kong Baptist University United International College, Zhuhai, China

**Keywords:** L2 learning experience, social contexts, identities construction, sustainable development of identities, poststructuralist framework

## Abstract

Informed by the poststructuralist theory, this study investigates the case of Ming, a Chinese professor of English, about the impacts of his first language (L1) and second language (L2) learning experience, and the changes of social contexts on his L1 and L2 identities construction. It was found that being a learner of English as a Foreign Language (EFL), Ming’s identities development aligned with the poststructuralist theory, in which it is considered dynamic, fluid and conflicting. Ming negotiated and renegotiated his identities in various social contexts in China and the United States and finally gained acceptance into the L2 academic community. This study not only analyzes Ming’s experience with his language learning and identities, but also unravels that conflicts may be part of the process of identities construction, and encourages learners to be persistent and emotionally resilient, while using certain strategies to retain a stable L1 identity so that they can navigate through the negative encounters during the second language acquisition (SLA) process to sustain the development of their identities and L2 abilities.

## Introduction

The relationships between social contexts, language and identities, and how they affect the learners’ second language acquisition (SLA) have been well documented in the literature. For instance, Schumann (1986) proposed the acculturation model, which asserts that ultimately various social and psychological factors in SLA lead to acculturation to the second language (L2) community. It anticipates that “the learner will acquire the second language only to the degree that he acculturates” (p. 379). Thus, in order for an individual to fully acquire an L2, he/she must be entirely adapted to the culture of his/her target language community, psychologically and socially. The English learner must be socially assimilated to his/her new L2 community by frequently interacting with his/her target language group. The learner must also be psychologically open to the new culture, adopting the new lifestyle and values of his/her L2 community. Such a theory essentially suggests that learners must decide whether they choose to retain their own heritage and culture, or to discard it to adopt the new culture. Choosing the former means that the learners will create social and psychological distance with his/her L2 community and will fail to acquire the L2. In other words, the acculturation model argues that for one to successfully acquire the second language, he/she must give up his/her first language (L1) culture to achieve social and psychological proximity with his/her L2 community.

Traditional frameworks such as the acculturation model embraced the monolingual model, which unrealistically oversimplifies the process of L2 learning and disregarded the learners’ heritage language and culture. However, the exponential increase in mobility and globalization in the last two or three decades has resulted in greater diversity in various communities. English as a lingua franca also seems to gain its momentum, and many people from non-English speaking countries began to learn English. The monolingual model can no longer reflect the realities of the world today. As such, more recently, several researchers argued against these frameworks with the poststructuralist model, which captures the complexity of L2 learning and renders the learning outcome unpredictable. For example, Norton (2010) described the poststructuralist framework and maintained that the linguistic communities may be diverse and conflicting, as the speakers negotiate and renegotiate their identities through using language in various social contexts, where power inequality prevails. This paper aims to add to the body of literature that supports the poststructuralist framework, by analyzing a case study of an advanced adult learner’s L2 learning experience to gain insights into how the changes of social contexts impact his L1/L2 identities construction. Drawing pedagogical implications from this case study, we hope to reduce the challenges of other L2 learners, and encourage them to strive through the difficulties, to make informed decisions about the development of their L1 and L2 identities, and to motivate all stakeholders (such as students themselves, parents, teachers, administrators in China and in the United States) to develop intercultural sensitivity necessary to support students’ L2 learning.

Based on the previous literature, there seems to be a need for more research on the complexity of how L1 and L2 learning affect the learner’s identities construction. In addition, there is a paucity of studies on the development of the learner’s identities associated with changing social contexts, which can be operationally defined in this study as the different residence countries, school settings, communities, circles of friends and family of an English as a Foreign Language (EFL) learner.

In order to better understand L1 and L2 identities construction along this line of research, the current study examines the case of Ming, a Chinese professor of English, about his L1 and L2 learning experience, L1 and L2 identities construction, and how language related experience and social contexts positively or negatively impact both identities.

## Literature Review

The theory of poststructuralism has been widely accepted in the academic community. Morgan (2007) explains that poststructuralists look at language as a tool to differentiate or categorize identities such as gender and ethnicity, which are inherently “multiple, contradictory, and subject to change across settings and through interaction” (p. 949). In addition, power always comes in between language/identity relations, and thus may bring about marginal positions. In addition, Norton (2006) summarized the five main characteristics of sociocultural studies (for example, Kanno, 2003; Pavlenko, 2004): (1) Identity, based on sociocultural theory, is dynamic and changes both in time and in space; (2) An individual’s identity is “complex, contradictory and multifaceted” (p. 24); (3) Identity develops and is developed by language; (4) the development of identity need to be understood through the notion of power, which may either be hostile or cooperative; and (5) The theory of identity is connected to classroom teaching. More recently, Norton and Toohey (2011) characterized identity as “fluid, context-dependent, and context-aims producing, in particular historical and cultural circumstances” (p. 419). Therefore, employing the poststructural and sociocultural framework, the present study of L2 identity (or learner identity) follows Norton (2000) to define identity as “the way a person understands his or her relationship to the world, how that relationship is constructed across time and space, and how the person understands possibilities for the future.”(p. 4)

Previous empirical studies substantiated the poststructuralist framework in learner identity and learner identity construction. Peirce (1995) undertook a longitudinal study regarding the identity construction of immigrant women in Canada, and proposed the concept of investment to capture the complexity of power relations, identity and language learning. In addition, she examined elementary students in Pakistan to illustrate that identity is a sociocultural construct and the students’ goal of English learning is to become legitimate members of the international community to access its privileges and resources (Norton, 2006). One of the important findings of the study is that the students’ English learning did not threaten their investment in their official language, Urdu. This suggests that English learning and L2 identity construction may be independent of the learners’ investment and identity construction in their home language.

Although English learners’ L2 identity construction may be independent of that of their official language in their home country, the process of their L2 identity construction may be difficult and complex. Therefore, a number of researchers conducted case studies investigating how L2 students negotiate their identities in an attempt to gain access to their L2 community. McKay and Wong (1996) conducted research on Chinese immigrant students in California and found that the learners negotiated their identities in multiple social interactions, where their identities were multiple, dynamic and at times conflicting. The students’ negotiations of identities were related to their practice of agency, which pertained to their positioning of power in school and community. Likewise, Morita (2004) case studies explored how L2 students in a Canadian university negotiated their “participation and membership” in the context of their L2 classrooms. It was found that learners struggled to negotiate their identities, competence and power positions in such context. Using the theory of second language of socialization, Ortactepe (2013) examined a Turkish doctoral student’s identity construction, which was marked with conflicts and struggles in gaining networks and access to the L2 community. Even though some L2 learners may struggle with their identity construction, it does not mean that they will not be accepted by their L2 community. Giroir (2014) reported her research on two Saudi Arabian males negotiating their identities in the United States post 9/11 contexts to successfully achieve higher participation in the L2 communities.

In more recent literature, scholars continue conducting similar studies, or updating their theories to gain a deeper insight into English learners’ L2 identity construction, especially how L2 learners negotiate their identities, the techniques they use and how they may mitigate such struggles. Darvin and Norton (2017) outlined the relationship between investment and research in language education globally that included a recently developed model, in which investment is linked to ideology, capital and identity. This model acknowledges the ease of teachers and students to move across time and space in the digital era. Moreover, Alali et al. (2020) examined the identity construction of L2 English learners in a university in Jordan. The findings showed that the learners were using numerous linguistic techniques to construct their identities, and may resist the identities given by other team members if these identities were believed to be inappropriate. In a similar manner, Brunton and Buckley (2020) qualitative study explored the experience of 34 first-year English students in two higher education institutions in Ireland. The investigation lasted approximately 1 year and revealed that although the students seemed to struggle with their identities to various extents, it could be lessened if they had a stable sense of self. The findings of these more recent studies shed a positive light on the negotiation process of English learners’ L2 identities with the supporting role of their L1 identity. These studies illustrated that although this process may be challenging, there are techniques and practices that may mitigate such difficulties. One of these techniques is the sustainable development of their L1 identity, which the learners could rely upon.

The research conducted on learner identity and its development supported the advocacy of multi-competencies and multilingualism. Pavlenko (2002) advocated for equity in accessing language and educational capital, encouraging multilingualism, and argued against “acculturation” and/or “monolingualism.” Likewise, Blackledge and Pavlenko (2001) explored the values and practices of language use in multilingual contexts, linking power relations and inequity to the beliefs about language use. In contrast, Kobayashi and Rinnert (2013) longitudinal case study examined how a Japanese multilingual writer advanced her Japanese, English and Chinese competency in writing. They found that “theories of multicompetence, genre, and identity” may enhance the multilingual’s unique writing style (p. 4).

Furthermore, Carter (2017) undertook a narrative inquiry of a multilingual Japanese, Naoko, who was an autonomous language learner pursuing a TESOL degree in an English speaking country. There were a few lessons worth learning from the participant’s story. For instance, when Naoko was in the military base at the age of 15, she was no longer scared and anxious like her earlier encounters when she was a child, her experience there was that of a positive one. The primary reason is that the American military base was a friendly environment where she was engaged in English with her peers. Finally, Fisher et al. (2020) proposed a new theory for “participative multilingual identity negotiation,” which utilized some key parts of “psychosocial, sociocultural and poststructural approaches to identity” (p. 462). Similar to previous studies about L2 identity, challenges in the practices of language use in multilingual contexts seem to be inevitable, due to language hierarchy and inequity. However, we should not assume that multilinguals’ experience in multilingual and/or English speaking contexts is always negative and that they are unable to negotiate their identities. On the contrary, some contexts may facilitate multilinguals’ identity construction. In addition, researchers are developing new theories to help learners construct their multilingual identities. An example of these new theories is Aronin and Singleton (2012) Dominant Language Constellations model, which takes away the language hierarchy within the model to help students better understand their pluralist identities.

While previous studies unfolded the L2 learners’ dynamic negotiation of identities, L2 competence, and power positions in diverse contexts to gain legitimate membership in the L2 and international communities, the complexity of how L1 and L2 learning experience impact the construction of L1 and L2 identities, and how both identities are affected by social contexts, are yet to be fully explored. The current study aims to shed lights on these issues by conducting a case study on a highly proficient Chinese professor of English, Ming, who was born and raised in China but went to the United States for his MA and Ph.D. studies. The case study seeks answers to the following research questions:

1) To what extent did Ming’s L1 and L2 learning experiences affect his L1 and L2 identities construction in different social contexts of China and the United States?
2) How had Ming’s different social contexts in China and the United States impacted his L1 and L2 identities development?

## Materials and Methods

This study took a qualitative case study approach, with Ming as our focal participant. The case study approach was used in this research because of its many advantages. For example, Merriam (1998) points out that “a case study design was employed to gain an in-depth understanding of the situation and meaning for those involved. The interest is in process rather than outcomes, in context rather than a specific variable, in discovery rather than confirmation” (p. 19). Creswell (2019) states that a qualitative case study as “an in-depth exploration of a bounded system (e.g., an activity, event, process, or individuals) based on extensive data collection” (p. 485). Case studies have been used widely (see e.g., Bao et al., 2021; Soltani and Zhang, 2021; Sun and Zhang, 2021), including investigating identity change (Jiang and Zhang, 2021). For these reasons, we now proceed to provide details on the case participant, Ming.

### Ming’s Profile

Ming was born and raised in a southern province of China and excelled in his Chinese and English, from a young age. After graduating with a BA in English in China, he prepared for the TOEFL and the GRE on his own and achieved nearly perfect scores, with 110 on TOEFL (its scores range from 0 to 120) and 98th percentile on the GRE. He went to the United States and stayed there for 10 years to pursue his MA and Ph.D. degrees in English. Upon graduating from his Ph.D. program, he started teaching and conducting research at a university in China. His L2 learning experience and identities of L1 and L2 might have been impacted because of a change in social contexts from China to the United States in general. More specifically, his social contexts in this study encompass different countries, school settings, communities, the circle of friends and family, etc. Therefore, he was chosen for this case study. A summary of Ming’s profile is presented below.

### Data Collection and Analysis

Two different data sources, namely, Ming’s written autobiographical narrative and a semi-structured interview, were employed in this study. Both were done in English. Five guiding questions (see Supplementary Appendix A) were generated to guide his narrative writing. After that, interview questions (see Supplementary Appendix B) were developed to probe into his L1 and L2 learning experiences that impacted his identities construction in different contexts in China and the United States.

In order to investigate Ming’s L1 and L2 learning experiences and identities construction, his narrative data were analyzed chronologically, based on three critical time periods. Each of the time periods is of significance to his L1 and/or L2 identities development. The first period occurred in the context of a high school and a university in China, where his L1 and L2 learning experiences as well as L1 and L2 identities construction began. The second period impacted his L1 and L2 identities development as his social contexts changed, when he first arrived in a mid-west city in the United States for his MA study. This is the period when he experienced the most struggles with his L1 and L2 identities. In contrast, the third period witnessed how Ming mitigated such conflicts and how he further developed both of his identities during his Ph.D. study in a large metropolitan city on the east coast of the United States. To deepen the analysis and triangulate the data, follow-up interview data were transcribed, coded, analyzed and presented based on the three time periods in the findings as follows: (1) L1 and L2 learning experiences and related language identities construction in China; (2) Initial L2 learning experiences in a mid-west city of the United States and language identities struggles; and (3) Reconciling L1 and L2 identities during the Ph.D. program in a large city of the east coast of America. Moreover, the poststructuralist theory was used to provide a deeper analysis of this case study. Member check was conducted with the participant to ensure accuracy and validity of data analysis.

### Findings

It is necessary to analyze Ming’s English learning experiences from three different time periods chronologically in order to capture his changing L1 and L2 identities. Ming’s profile and English learning are summarized in Table 1. And changes in contexts and development of his L1 and L2 identities in the three time periods are summarized in Table 2.

**TABLE 1:** Ming’s profile.

Academic context	Duration	Content of learning	Evidence of high English proficiency
Chinese Public Schools	11 years	English as a subject	
Chinese University	4 years	English major	Official TOEFL score: 110 Official GRE score: 98 Percentile
MA in the United States	2 years	English	Published two journal articles in 2nd and 3rd semesters
Ph.D. in the United States	7 years	English	Continued to publish journal articles and books

**TABLE 2:** The relationships among Ming’s language learning, social contexts and identities construction.

Language learning	Social contexts	L1 and/or L2 identity construction
Chinese and English learning	A high school in China	Primarily L1 identity
English learning	A university in China	Primarily L2 identity
English learning	MA program in a mid- west city of United States	L1 and L2 identities were sometimes conflicting
English learning	Ph.D. program in an east coast city of United States	L1 and L2 identities co-existed and both were considered assets

#### Period One: L1 and L2 Learning Experiences and Related Language Identities Construction in China

As Ming grew up in a multilingual environment with exposure to different dialects, he considered himself a multilingual Chinese. It seemed that at that time, his L2 identity was much weaker as compared to his L1 identity, since it was only “a foreign language” to him. Nevertheless, his L2 identity developed from a beginner of English in junior high school to an advanced learner at university:

Excerpt 1 from narrative

“…*At the age of 11, I began to learn English as a foreign language in junior high school*…*I became an English major in college and studied the language, culture, and pedagogy of English. Apparently, I am a native speaker of Chinese*… *English is just a foreign language to me.”*

As an avid reader and skillful writer in his L1, Chinese, Ming believed that some of his L1 reading and writing skills were positively transferred to his acquisition of L2 English, which boosted his confidence in reading and writing in English and subsequently reinforced his L2 identity.

Excerpt 2 from narrative

*“My solid foundation in Chinese literacy contributed a lot to my acquisition of English literacy later on which increased my confidence in the English language immensely.”*

#### Period Two: Language Identities During the Master’s Program in the United States: Construction, Retainment and Conflicts

When Ming arrived in a mid-west city of the United States for his MA study, he still considered himself an English learner:

Excerpt 3 from narrative

*“I considered myself a lifetime English learner. In the MA program, I read on average 300 pages English materials per week, and delivered six to eight presentations and wrote four term papers (3,000 words each) every semester, plus a thesis in the 4th semester. Meanwhile, I was watching English TV and interacted with Americans on a daily basis. the language environment changed to English. most of my professors and classmates were Americans.”*

Excerpt 4 from interview

“…*my English improved a lot. In the second semester of my MA program, I wrote a critical and insightful book review in English and published it in a refereed journal. Then I published another paper in my 3rd semester. At that time, none of the American students in my MA cohort published any papers.”*

He was developing his L2 identity in the United States. Meanwhile, he reached out to various Chinese and American discourse communities, and retained his L1 identity.

Excerpt 5 from narrative

“… *However, my roommate was a Chinese graduate student and I called my family once a week. In addition, I regularly interacted with people in the Chinese community. I was able to retain my L1 self in the United States.”*

However, such developments of his identity were not without struggles and conflicts.

Even though he was able to gain acceptance into both the American and the Chinese communities in the United States, the communities themselves were separated from each other. He felt as if two people, namely, his L1 self and his L2 self, were trapped living within one body. Moreover, Ming also felt that his L1 identity threatened his L2 identity at times, and vice versa:

Excerpt 6 from interview

*“When Americans were hostile toward my L1 self, it impeded my L2 learning, because I felt like I was not eligible to speak English. One day, I was doing grocery shopping at a local supermarket in that mid-west city. I was talking to my Korean friend about some random stuffs. All of a sudden, an American guy walked by and said to me “You are Chinese, why do you speak English? That’s my language!” I did not confront him or argue with him. Somehow I felt that he owned the English language and I did not have the right to use it. It was a horrible feeling.”*

Another hurdle that Ming needed to overcome was the unfamiliarity of American culture, for instance, the background knowledge of jokes. A lot of the time, when the professors said something funny in class, he did not know what was going on when everyone else was already laughing out loud.

Excerpt 7 from interview

*“Sometimes, when talking to my American classmates, they were also joking too (in a friendly way, you know.), but I did not get the joke and had no clue what they were talking about. Those were embarrassing moments.”*

He also encountered problems when interacting with Americans.

Excerpt 8 from interview

*“Chinese people may say “have you eaten?” as a way to say “hello” to each other. Of course, I was not stupid enough to say that to greet my American friends. However, I was not used to saying “how is it going?” or “what’s up?” yet when I bumped into my friends in the street or on campus. Instead, I greeted them in the Chinese way by saying “where are you going?”. Guess what? Some Americans responded “none of your business.””*

Apparently they were offended by what he said. Americans seem to have a strong sense of boundaries between each other. They value privacy and try to mind their own business. He stumbled over cultural issues like that quite a bit.

On the other hand, after living in the United States for about a year, Ming began to encounter issues with some friends back in China. After he went to the United States for graduate study, he barely hand wrote Chinese anymore. As a result, his ability in Chinese writing decreased. Even though his everyday Chinese was not much affected, he sometimes had trouble expressing academic stuffs in Chinese.

Excerpt 9 from narrative

*“One day, I was talking on the phone in Chinese to a friend in China and he asked me about the format of a research paper. I could not think of the Chinese words for “double spaced,” so I just said the English words, which he did not understand. He was offended and screamed at me: “Why do you have to show off your English? Do you think you are all that?”. Things like that happened to me a few times.”*

Even though it was not his intention to say English words to his friends in China, his L2 identity still posed a threat to his L1 self and caused miscommunication with his Chinese friends.

#### Period Three: Reconciling L1 and L2 Identities During the Ph.D. Program in the United States

After the master’s program, Ming moved to a large city on the east coast for his Ph.D. degree. There, his academic English further improved. He was serving as a research assistant and later a teaching assistant at university where he interacted on a daily basis with students and colleagues. During his doctoral study, he published quite a few papers in international refereed journals.

He was also able to maintain his L1 identity, especially when his American undergraduate students and his American colleagues highly respected him and his L1 identity, and welcomed him into the academic community. Perhaps it was because the university was located in a metropolitan city with a quite diverse population, people tended to embrace diversity more, and incidents like the one in the supermarket did not happen to Ming again.

Excerpt 10 from narrative

*“For some reason, some of my American undergraduate students and professors in the doctoral program preferred to call me by my Chinese name Ming rather than my English name Tom. I felt that they respected me for who I am and valued my Chinese cultural background.”*

His confidence in his L2 continued to grow as he published more journal articles and excelled in his doctoral study. He finally found a way to accept both his L1 and L2 identities, and became a multilingual, multicultural individual.

Excerpt 11 from interview

*“In the Ph.D. program, I got straight A’s. I remembered a professor of the Ph.D. program was a well-known tough grader. He rarely gave A’s to his students and his tough feedback even made some doctoral students cry. I survived his class and got an A for active participation in class discussions and writing compelling term papers.”*

When people doubted his L2 identity, he seemed to be able to fall back on his L1 identity, which was more stable and comforting to him. His knowledge about World Englishes also helped.

Excerpt 12 from interview

*“Sometimes people in my neighborhood picked on my Chinese accent and said that You don’t have a perfect American accent. How can you teach English then?”*

Ming was bothered by those negative comments at the beginning, but then as he read more literature on World Englishes, he knew well enough to tell those people that the monolingual model of English learning was problematic and unrealistic. In addition, as a lingua franca, English is being used world wide, instead of by people from English speaking countries only. Different varieties of Englishes are being redefined by researchers. His Chinese accent just shows that he was speaking a variety of English, which he was feeling comfortable with.

Furthermore, Ming was inspired by some well-established scholars in his field, who originally came from Asia but were working in the United States. They shared similar cultural backgrounds and probably went through similar struggles to fight prejudice as well.

Excerpt 13 from interview

*“In addition*…*there were quite a few Asian researchers*…*(who) were leaders in the field. I looked up to them as my role models and thought that my Chinese accent did not matter as long as I kept publishing papers and that should not be an obstacle to my career. Instead, my L1 self and culture were an asset in my research and teaching*.”

Finally, Ming no longer thought his L1 and L2 identities were conflicting. Instead, both were assets.

Below is a summary table of Ming’s L1 and L2 identities construction in different social contexts, reflecting the intricate relationships among his language learning, social contexts and identities construction.

## Discussion

Ming considered himself a life time English learner, and kept expanding his L2 identity in these three critical periods of his L1 and L2 learning. In the beginning, Ming’s L1 and L2 identities developed separately in China. Although his identity became a struggle when Ming first arrived in the mid-west of the United States for his MA study, during his Ph.D. study, his L1 and L2 identities converged and he was finally feeling comfortable with his identity as a multilingual. Ming was an additive bilingual. He moved to the United States after he graduated from university in China. By that time, his L1 identity was already fully developed. He managed to retain his L1 identity throughout his stay in the United States. Thus, his L2 identity was additive to his L1 self.

The construction of Ming’s L1 and L2 identities can be explained by Norton’s poststructuralist theory. His identities were fluid, dynamic and changed depending on the contexts. For example, during Ming’s MA study, his L1 and L2 identities were dynamic and fluid. When he was in school, he may present his L2 self to his professors and peers. However, when he was at home or talking to his family in China over the phone, his L1 self dominated. The struggles and challenges Ming went through were not only due to cultural gaps between the east and the west, it was also because of the power relations. For instance, when Ming could not think of the Chinese word for “double spaced,” he spoke English to his friend in China (who didn’t speak English), which made his friend angry. This is mainly because English is at the top of the language hierarchy, and the fact that Ming spoke English unintentionally put his friend at a disadvantage. It was after going through different changes in contexts and difficulties that Ming successfully developed his L2 identity.

Other findings show Ming had much positive L1 transfer to his L2 literacy, hence he looked at his L1 identity positively. Such may be due to his social context (a high school in China) at the time that allowed him to construct his L1 identity with ease. Furthermore, it seemed that Ming’s L1 identity conflicted with his L2 self after he went to the United States for his MA study. This was due to his social contexts at the time. He was studying in an MA program in a mid-west city in the United States. The culture in the mid-west is relatively conservative, being less liberal than that in the west and east coasts. Even though Ming was well accepted by both the Chinese and the English communities at that mid-west university [this is consistent with Pennington (2018), who maintained that people may have the benefit of more than one linguistic identity and gain access to more than one community], they were largely separated from each other. As such, Ming was never fully accepted by both communities since only one of his identities was recognized at a time. Moreover, some individuals in the English community not only rejected his L1 identity, but also expressed hostility toward it. For instance, the American man in the supermarket did not think Chinese are eligible to speak English because they do not own that language. This kind of negative experience may sometimes result in his weaker L1 identity (in order for him to gain acceptance into his L2 community) and impeding the development of his L2 self.

Ming’s struggles in his L2 identity are consistent with the previous studies (e.g., McKay and Wong, 1996; Morita, 2004; Ortactepe, 2013; Zhang and Zhang, 2015). Specifically, identities are socially constructed through the relationships and interactions between an individual and people around that person in specific contexts. The English learner struggles to negotiate and renegotiate his/her identity, and to gain access to the L2 community and networks. On the other hand, these struggles may be mediated through accomplishments in his/her L2 learning. In Ming’s case, he regained some confidence in his English learning through publishing in international journals.

This is similar to the research conducted by Alali et al. (2020), and Brunton and Buckley (2020), who observed that English learners may use linguistic techniques or depend on their accomplishments to construct their L2 identities and may resist the identities given by others. The students’ struggles in constructing their identities may be mitigated when they have stable selves, which largely rely upon their L1 identity. In addition, Ming’s academic success during his MA studies, which helped him develop a more stable L2 self, encouraged him to invest in further studies in the United States. This confirmed with Darvin and Norton (2017) model, in which investment is connected to, in this case, identity. Moreover, it is consistent with Takkaç (2019) findings, which illustrated that the process of English learning impacts the growth of one’s identity. These previous studies illustrated the possibilities of remedies against the learners’ challenges, which is what Ming experienced in this case study. Ming’s difficulties in his L2 identity construction were reduced because of his new found confidence, based on his achievements in successfully publishing papers in decent journals while he was only an MA student.

Moreover, Ming’s Ph.D. study and continued publications (an example of Ming expanding his L2 identity from an L2 learner to an international researcher) helped him gain confidence in his English and L2 identity. The fact that his professors, and peers valued his diverse background helped to nurture his identity as “a multiple and a dynamic social construct” (Norton, 2010, p 56). He also found role models who were international researchers originally from Asia with similar backgrounds and experience. Compared to his MA studies, it seems that Ming was in a more supportive and diverse environment during his Ph.D. study, where the English community accepted both his L1 and L2 identities. This had a huge impact on Ming’s L1 and L2 identities. Specifically, both identities co-exist without being in conflict with each other. Furthermore, he acknowledged the benefits of being a multilingual, and became a multicultural person, where his intercultural sensitivity may be improved due to others’ respect for his identities. This is consistent with Carter (2017) about the narrative of a multilingual Japanese Naoko. Both Ming and Naoko had positive experiences because of the supportive environments, which helped them with their English learning and learner identities. In addition, as a result of Ming’s positive experience, he may have become more open to new ideas, more tolerant and embracing people from different cultures or other backgrounds, and highly value the unique perspectives that they bring to the academic community. The fact that Ming was eventually accepted by his L2 community and networks is consistent with the findings of Giroir (2014). Both Ming and the two Saudi Arabian males have obtained higher participation in their L2 communities. Such clearly suggests that success in the learners’ L2 identity construction and memberships is possible and attainable.

Furthermore, Ming was raised in a multilingual environment with exposure to different dialects. Consequently, he thought of himself as a multilingual, which may have enabled him to maintain such an identity in the United States, even when his L1 and L2 identities conflicted with each other. Contrary to Schumann (1986) acculturation theory, Ming achieved incredibly high proficiency in English without being fully acculturated. When he was in the United States, he enjoyed interacting with his fellow Chinese expatriates and communicating with his family in China regularly over the phone, whom he “*held dear*” in his “*heart.*” He also expressed that his “*favorite food is still Chinese dishes.*” While existing studies promote multilingualism, and advocate against acculturation and monolingualism, it is essential to point out that although Ming was not fully acculturated, he became more inter-culturally sensitive, and embraced diversity. This implies that although English learners do not have to fully accept the L2 culture and lifestyle, it may be beneficial for them to have an open mind and to be more inter-culturally sensitive, so that they may interact with their L2 communities easily.

Ming’s autobiographical narrative and interview data seemed to align with the conceptual framework of the poststructuralist theory, described by Peirce (1995), Norton (2010). Ming’s identities were complex and dynamic. He struggled with his L1 and L2 identities and negotiated and renegotiated them in various discourse communities. In the supermarket, Ming was questioned about his eligibility to speak English because he was Chinese instead of American; in the local community, his Chinese accent was criticized; on campus, he stumbled multiple times for not being familiar with American culture; he even ran into problems with some friends back in China while talking on the phone and using a few English words. Such encounters were mostly because of the higher status of the English language (power) that created inequality in the diverse United States society. The monolingual model of language education and the prevalence of language hierarchy in the society have been challenged by various researchers. The dichotomy of non-native versus native English speakers stigmatizes those who speak English as an additional language, negatively impacts their confidence in using English and hinders their English language learning (e.g., Shibata, 2021, cited in Xiao et al., 2021). However, we need to be cautious not to invert the power pyramid, where people who were once being dominated at the bottom are discriminating and prejudicing against those who were dominating at the top (Wu and You, 2018, cited in Xiao and Zhao, 2021).

Furthermore, it is equally important to recognize that not all discourse communities augment Ming’s struggles with his identities. Some (for example, the communities during Ming’s Ph.D. study) may nurture and fast-track his L2 identity construction and encourage both of his L1 and L2 identities to co-exist and thrive.

## Implications

Ming’s experience revealed important implications on English learners’ identity construction and sustainable development. First, there may be a relationship between students’ L1 and L2 identities and L2 learning. Various researchers studied the socio-contextual framework of L2 learning (for example, Noels and Clément, 1996, cited in Rubenfeld et al., 2006, p609), and found that one’s L2 learning is affected by his/her interaction with “the L2 community, L2 confidence and identification to both the first language and L2 community” (Clément, 1980; Noels and Clément, 1996, cited in Rubenfeld et al., 2006).

Therefore, in order to facilitate students’ L1 and L2 identities construction and sustain their development, teachers should respect students’ diverse backgrounds, create a non-threatening environment and be sensitive to students’ diverse needs. For example, as their L2 identity is not strong enough to enable them to enter into the L2 community, some learners may rely upon their L1 identity when they encounter adverse situation during the process of learning L2 and joining the L2 community. Ming is a case in point. Secondly, there are discourse communities that facilitate English learners’ L2 identity construction and encourage the sustainable development of multilingualism and multiculturalism. Individuals in these communities will feel welcomed and accepted, and may construct their identities without having to overcome as many challenges and struggles. In the particular case of Ming’s experience in the United States, the mid-west city was less liberal than the east coast city; the local community was less open-minded than the researchers’ community; and the MA program was less supportive than the Ph.D. program.

Third, the learners need to build up emotional strengths to deal with negative experiences that come with their L1 and L2 identities, as struggles and conflicts may be inevitable depending on their social contexts. The constructing, reconstructing and expanding of identities may be mediated by the negotiation between the identities and a wide array of positive and negative emotions that occur in the complex sociocultural context (Wu et al., 2021). Lastly, even in adverse situations and contexts, learners may still be able to sustain the development of L1 and L2 identities through mediating the struggles and making progress through achievements (for example, academic success).

## Conclusion

The identity development of a multilingual/bilingual does not seem to be easy, and its success may depend on the individual’s social contexts. This study presented three critical periods of Ming’s L1 and L2 learning experiences that profoundly impacted his L1 and L2 identities development. It was found that Ming’s identities development is mostly consistent with the poststructuralist framework, in which it is considered dynamic, fluid and conflicting. Ming negotiated and renegotiated his identities in various social contexts and finally gained respect from the L2 academic community.

This line of research could be pursued further. To begin with, Ming’s identity is dynamic with multiple dimensions. Other than being an English learner, he was also a teacher and a scholar, both of which could become a focus of future research. And insights into his professional identity construction could be intriguing (Wang and Zhang, 2021). As the purpose of this article was to investigate the process of Ming’s L1 and L2 identities construction and development, it only focused on his L2 learning, social contexts and L1 and L2 identities. The topics could be expanded. For instance, future research could probe into the connection between Ming’s L2 motivation and his identities construction. In addition, a longitudinal study may allow Ming’s English competence development to be unfolded more fully and observed more closely. Due to the time constraint, the current study only relied on one participant’s retrospection on his past experience related to L1 and L2 learning and identities construction instead of data collected over an extended period of time.

Lastly, the present study is a unique case, and such studies of multilinguals/bilinguals should be done on a case by case basis, in order to capture the complexities (especially in terms of the ages and contexts) of the learner identities construction. There may be others who may have different experiences in their English learning and their identities construction. For example, some may have moved to an English speaking country at a younger age, at an older age, or may be born in that country. Some learn L2 in an L2 speaking context whereas others may learn it in an L1 environment. Nevertheless, they are also multilinguals/bilinguals who may have a unique point of view on various pertaining topics. Therefore, it may be important for one to acknowledge, respect and embrace such diversity, instead of giving preference to one “type” of multilinguals/bilinguals. Findings of various studies (including studies on larger scale multiple cases) may offer empirical data to substantiate or challenge existing theories, or develop new theories and techniques to alleviate the challenges in students’ L2 identity construction, and contribute to the sustainable development of students’ L2 skills.

For the above reasons, more empirical research (case studies and survey plus interviews) should be conducted to provide stakeholders with a more realistic and panoramic picture that reflects the complexities of L2 learners identities construction, the potential opportunities and the role of learner agency as a result of sustaining the development of their L2 identity and creating their multilingual identity.

## Data Availability Statement

The raw data supporting the conclusions of this article will be made available by the authors, without undue reservation.

## Ethics Statement

The studies involving human participants were reviewed and approved by the Foreign Studies College, Hunan Normal University. The patients/participants provided their written informed consent to participate in this study.

## Author Contributions

YX was responsible for the conceptualization, most of the methodology and editing. AZ contributed to the first draft of the manuscript. Both authors contributed to the data collection, revising, and approved the submitted version.

## Conflict of Interest

The authors declare that the research was conducted in the absence of any commercial or financial relationships that could be construed as a potential conflict of interest.

## Publisher’s Note

All claims expressed in this article are solely those of the authors and do not necessarily represent those of their affiliated organizations, or those of the publisher, the editors and the reviewers. Any product that may be evaluated in this article, or claim that may be made by its manufacturer, is not guaranteed or endorsed by the publisher.
