# Supplementary material for: A Case Study on the Impacts of Social Contexts on a Chinese English as a Foreign Language Learner’s L1 and L2 Identities Development
Source: Front Psychol. 2022 Jan 6;12:772777. doi: 10.3389/fpsyg.2021.772777 (PMC8770975; doi:10.3389/fpsyg.2021.772777)
Supplement: Supplementary file 1 [file Data_Sheet_1.docx]

**Appendix A**

Guiding questions for narrative inquiry:

1. What were your language environment and language learning experiences in China?

2. How did your language environment change after you moved from China to the US?

3. How did you learn L2 (English) in the US?

4. How did the change of your language environment impact your L1 (Chinese) self and your L2 self respectively?

5. How did your L1 self and L2 self impede or facilitate your L2 learning?

**Appendix B**

Interview questions:

1. How did you feel about yourself as an English learner when you first arrived in the US?

2. Did you feel that you have advanced as an English learner in the US? How?

3.After you went to the US, did your use of English affect your use of L1?

4. How did you feel when you spoke L1 in the US?

5. How was it different with that in China?

6. Did you encounter any trouble when you used L1 or L2 after you went to the US? How?
